# Manual traction is effective in alleviating lumbosacral spine pain: Evidence from a randomized controlled trial

**DOI:** 10.1016/j.heliyon.2024.e31013

**Published:** 2024-05-10

**Authors:** Rafał Studnicki, Piotr Szymczyk, Tomasz Adamczewski, Karolina Studzińska, Rita Hansdorfer-Korzon, Ana Filipa Silva, Adam Kawczyński

**Affiliations:** a Sub-Faculty of Physiotherapy, Medical University of Gdańsk, Gdańsk, Poland; b Medical Rehabilitation Clinic, Chair of Rehabilitation, Faculty of Health Sciences, Medical University of Lódź, Łódź, Poland; c Escola Superior Desporto e Lazer, Instituto Politécnico de Viana do Castelo, Rua Escola Industrial e Comercial de Nun’Álvares, 4900-347 Viana do Castelo, Portugal; d Sport Physical Activity and Health Research & Innovation Center, Viana do Castelo, Portugal; e Wrocław University of Health and Sport Sciences, Departament of Paralympic Games, Wrocław, Poland

**Keywords:** Low back pain, Traction, Physical therapy modalities, Pain measurement, Randomized controlled trial, Manual traction, Pain assessment tools

## Abstract

**Background:**

Manual traction, a therapeutic technique frequently employed in healthcare, involves applying controlled pulling force by hand, usually to the spine, to stretch muscles and decompress joints, thereby alleviating pain. This method can be particularly beneficial for addressing lumbosacral spine pain exacerbated by radicular symptoms, characterized by pain radiating from the lower back due to compression or irritation of spinal nerves.

**Purpose:**

This study aimed to compare the effects of manual traction against control group in alleviating the lumbosacral spine pain caused by radicular symptoms.

**Methods:**

A randomized controlled study design was utilized with a sample of 60 patients experiencing lumbosacral spine pain, evenly distributed between an experimental group (n = 30; receiving manual traction) and a control group (n = 30). Patients underwent assessments before and after six treatment sessions, which included the Straight Leg Raise test, modified Bragard's test, Kernig's test, and the visual analogue scale for pain perception.

**Results:**

Between-group significant differences were found at post-intervention, favoring the experimental group on SLR – Left (°) (p = 0.004; medium effect size), SLR – Right (°) (p = 0.004; medium effect size), Modified Bragard test – Left (°) (p = 0.024; small effect size), Modified Bragard test – Right (°) (p = 0.003; medium effect size), Kernig's Test – Left (°) (p = 0.013; medium effect size) and Kernig's Test – Right (°) (p = 0.010; medium effect size). Additionally, between-group significant differences were found at post-intervention, favoring the experimental group on VAS scores at SLR left (p < 0.001; medium effect size), and right (p < 0.001); medium effect size, Modified Bragard test left (p < 0.001; medium effect size) and right (p < 0.001; medium effect size) and at Kernig's Test left (p < 0.001; medium effect size) and right (p < 0.001; medium effect size).

**Conclusions:**

In conclusion, manual traction is recommended as an effective approach for alleviating lumbosacral spine pain in patients experiencing symptoms resulting from irritation or compression of a spinal nerve root.

## 1 Introduction

Radicular syndromes, characterized by pain, sensory deficits, and motor dysfunction along specific nerve root distributions, result primarily from anatomical abnormalities in the spine [1]. Disc herniation, spinal stenosis, and vertebral fractures are common culprits, directly compressing or irritating nerve roots [2]. Labor-intensive work can exacerbate these conditions by subjecting the spine to repetitive stress and improper loading, accelerating degeneration and increasing the risk of nerve impingement [3]. Conversely, the prolonged periods of sedentary behavior, such as office work involving long hours at a computer, may contribute to the development or exacerbation of radicular syndromes [4]. While not the sole cause, sedentary lifestyles and prolonged sitting have been linked to various musculoskeletal issues, including low back pain and radicular symptoms [5]. Prolongated physical inactivity weakens spinal support structures, predisposing individuals to spinal instability and exacerbating existing pathologies [6].

Radicular syndromes, such as sciatica, are widespread globally, impacting a substantial segment of the population [2]. A retrospective study involving 1669 participants revealed that 28.1 % of individuals displayed spinal disorders [7]. Among these, the lumbar spine (53.1 %) and cervical spine (27.1 %) were the most frequently affected regions, with pain emerging as the predominant complaint. Similarly, another study involving 34,902 individuals [8] found that low back pain was the most prevalent, followed by neck pain and thoracic pain. Incidence typically rises with age, reaching a peak during the fifth and sixth decades of life, although it can also affect younger individuals [7]. Pain associated with radicular syndromes ranks as one of the most common cause of intermittent work disability [9].

Among patients experiencing radicular pain, only 5–10 % of those with herniated lumbar discs necessitate neurosurgical intervention, while the majority opt for conservative treatments like pharmacotherapy, spinal orthoses, or manual therapy, particularly traction [[10], [11], [12]]. Manual traction is a therapeutic approach frequently employed to alleviate radicular pain, arising from compression or irritation of spinal nerve roots [13]. Scientific evidence suggests that manual traction offers benefits by decompressing affected spinal segments, thus reducing pressure on the nerve roots and alleviating pain [[14], [15], [16]]. Specifically, lumbar traction may help reduce pressure on the vertebral foramen by releasing tension between adjacent vertebrae. Additionally, it may alleviate muscle tension around the affected area or decrease intervertebral pressure, potentially aiding in the retraction of the protruding nucleus pulposus within the disc [[17], [18], [19]].

Despite the potential benefits of manual traction in alleviating this pain, its utilization is not widespread. For instance, in Northern Ireland, only 15 % of patients receive traction, compared to 41 % in the UK, and a much higher percentage, ranging from 58 % to 77 %, in outpatient rehabilitation physiotherapy providers in the USA [20]. The difference in usage can be attributed to various factors, including differing beliefs about its effectiveness. However, a recent systematic review with meta-analysis demonstrated that manual traction significantly reduces pain and disability in the short term [21].

For instance, lumbar extension traction combined with other therapies provides better outcomes in pain, disability, and intervertebral movements for patients with discogenic lumbosacral radiculopathy compared to control groups [22]. Also, side-lying manual lumbar traction can significantly reduce pain during active lumbar motion in patients with lumbar disc degeneration [23]. However, the effectiveness of manual traction may vary depending on the specific spinal condition and the presence of other therapeutic interventions [24].

While studies on manual traction are well-established [15,21], there is a scarcity of research specifically focusing on lumbosacral spine pain with radicular symptoms. Addressing this critical gap in the existing literature concerning the efficacy of manual traction in managing lumbosacral spine pain with radicular symptoms is imperative. This endeavor not only aids in comprehending the safety and effectiveness of manual traction but also provides scientific evidence to guide practitioners in implementing evidence-based strategies. As a technique potentially alleviating symptoms, this is particularly relevant for mitigating pain in patients while employing appropriate strategies.

Additionally, conducting a comprehensive pain screening using tests such as the Straight Leg Raise test (SLR), Modified Bragard's Test, Kernig's Test, and Visual Analogue Scale (VAS) can offer a comprehensive understanding of how manual traction impacts pain experienced by patients under various conditions. Conducting a comprehensive assessment using all these tests, provides a holistic understanding of how manual traction affects patient pain under various conditions, with SLR evaluating sciatic nerve irritation or disc herniation, Modified Bragard's reproducing symptoms for nerve root evaluation, Kernig's assessing for meningitis-related stiffness, and VAS quantifying subjective pain experiences for standardized monitoring and treatment efficacy assessment. With this in mind, the present randomized clinical study aims to compare the effects of manual traction against a control group in alleviating lumbosacral spine pain attributed to radicular symptoms.

## 2 Methods

### 2.1 Study design

This study adopted a randomized controlled design. We employed convenience sampling and utilized various recruitment methods, including announcements, individual contacts within specific labor organizations, and public notices on social media platforms. The randomization process involved opaque envelopes, randomly assigned to participants, ensuring allocation concealment prior to baseline assessments. Furthermore, the researcher responsible for ensuring eligibility criteria remained blinded to the randomization and allocation processes, thereby safeguarding the impartiality of decisions regarding subject allocation to specific groups. Assessors at baseline and post-intervention were blinded to group assignments. Between the two evaluation points, the experimental group (exposed to manual traction) underwent six intervention sessions, while the control group received a different type of physical therapy. The study was conducted until June 2020 at the CTM - Manual Therapy Centre in Malbork, in collaboration with the Sub-Faculty of Physiotherapy at the Medical University of Gdańsk.

### 2.2 Ethical aspects

The study design received approval from the Ethics Committee of Wrocław University of Health and Sport Sciences (NKBBN1/2023) and adhered to the current ethical standards in Exercise Research outlined in the Declaration of Helsinki. Participants were briefed on the study's design, associated risks, and potential benefits. Upon providing consent, they signed a free consent form clearly indicating their option to withdraw from the study at any time without penalty. Thus, written inform consent was provided. Furthermore, participants consented to the publication of their data anonymously.

### 2.3 Participants

The initial sample size was estimated a priori to achieve a power of 0.85, with a significance level of 0.05, considering two groups and two measurements, and an effect size of 0.4. This estimation conducted using G*Power software (version 3.1.9.6., Universität Düsseldorf, Germany), yielded an estimated number of participants of 46. The inclusion criteria for participation in the study were as follows: (i) reporting low back pain and diagnosis confirming radicular conflict with no causes other than radiculopathy; (ii) participating in both assessment moments, (iii) attending all therapy sessions; (iv) > 18 years old; (v) no comorbid diseases (e.g., tumour, infection, rheumatoid arthritis, severe vascular disorders, strains and sprains, inflammation and instability). The exclusion criteria for participation in the study are as follows: (i) individuals who do not report low back pain or lack a diagnosis confirming radicular conflict; (ii) those unable to participate in both assessment moments or attend all therapy sessions; (iii) participants under 18 years old; and (iv) individuals with comorbid diseases such as tumors, infections, rheumatoid arthritis, severe vascular disorders, strains and sprains, inflammation, or instability.

**Fig. 1:**
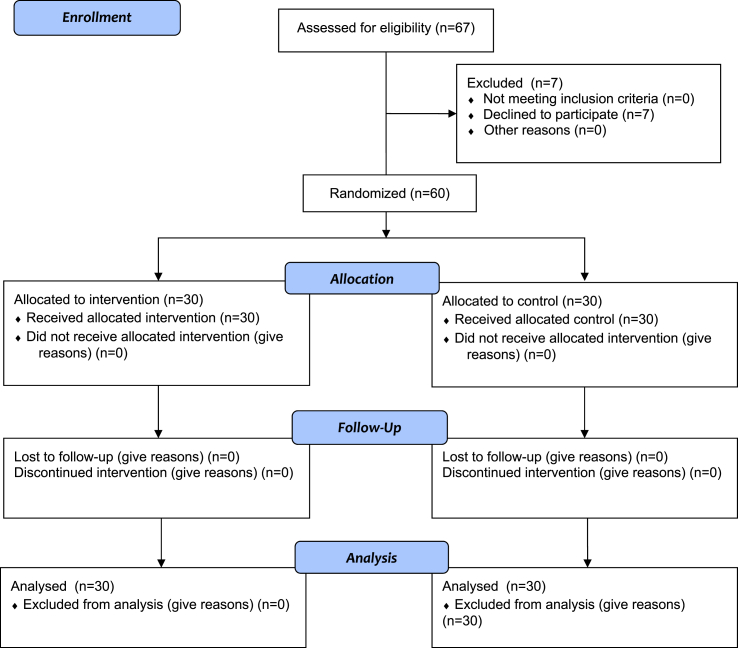
Participants flowchart.

Following the recruitment process, 67 individuals expressed interest in participating. However, after receiving detailed information about the study design and timeline, 7 individuals withdrew their participation. Subsequently, the remaining 60 participants were randomly assigned to either the experimental or control group (Fig. 1).

The participants had an average age of 48.0 ± 8.7 years, comprising 29 men and 31 women. The experimental group had an average age of 47.7 ± 9.4 years, with 15 men and 15 women, while the control group had an average age of 48.4 ± 8.1 years, with 14 men and 16 women.

All subjects were assessed based on medical diagnoses derived from imaging examinations (CT, MRI) and medical history. Within the experimental group, 10 patients were diagnosed with protrusion, 3 with hernia, and 17 with discopathy. In the control group, 11 had protrusion, 6 had hernia, and 13 had discopathy.

### 2.4 Experimental approach and therapies

Both the experimental and control groups underwent a two-week therapy program, which included diadynamic currents (6 min), light therapy using a Sollux lamp (15 min), laser therapy (3 min), and magnetotherapy (15 min). Each physiotherapeutic procedure was administered ten times. In addition to the standard therapy regimen, the experimental group received six additional therapy sessions exclusively focused on manual traction on alternate days. The control group did not receive any manual traction sessions.

### 2.5 Methodology of the manual traction performed

In the experimental group, 10-min traction was applied to the lumbosacral spine in the supine position, with the lower limbs bent at the hips, knee joints at a straight angle, and the feet resting on the therapist's chest. The upper limbs were arranged along the length of the body, and the head was in extension of the body. The patient, in the supine position, was stabilized on the therapeutic table with a belt, at the level of the rib arches. The therapist was positioned at the end of the therapy table. The traction belt covered the proximal part of the patient's thighs and the therapist's pelvis. The therapist, by shifting his body weight to the back leg and, at the same time, shifting his torso backward, transferred the thrust via the traction belts onto the lumbosacral spine.

The therapist increased the thrust until the patient began to feel pain rated two on the VAS scale or reported feeling stretching in the paraspinal soft tissues of the lumbosacral segment. The therapist maintained the prescribed thrust force until the patient no longer felt pain or the stretching of tissues. Then, the therapist increased the thrust until the pain rated two on the VAS scale reappeared, or the feeling of stretching the tissues returned, and again maintained the set constant thrust until the patient felt no pain or reported that he didn't feel the stretching of tissues. After the patient confirmed that he no longer experienced pain or tissue stretching, the therapist once more increased the thrust force until the patient again felt pain rated two on the VAS scale or reported the sensation of the tissues being stretched. The therapist maintained the prescribed constant thrust until the sensation of pain or tissue stretching disappeared.

After performing three series of increasing thrust, the therapist reduced the thrust by thirty percent and maintained the prescribed thrust at a constant level for approximately thirty to 40 s. A thirty percent reduction in thrust allowed the soft tissues to rest and was the baseline thrust for the successive phases of thrust increases. The therapist performed three progressively changing tractions, let the soft tissues rest, and then performed two more such series of progressive thrusts. If the pain on the VAS scale in the lumbosacral region persisted or increased due to the constant thrust, it was reduced. After the traction was performed, the patient rested for 20 min on the therapy table in a supine position with the lower limbs bent at the hip joints about forty-five degrees, feet positioned on the therapeutic table, resting on their plantar part.

Thus, in summary, one session lasted for 3 min and was divided into three parts, each lasting 1 min. During each 1-min interval, traction was applied for 45 s, targeting a pain sensation level of 2–3 on the VAS scale for the patient. This was followed by 45 s of relaxation, allowing the patient to return to the starting position. After each 3-min session, there was a 30-s break, and this sequence of 3-min sessions was repeated three times.

### 2.6 Assessments

The participants underwent assessments twice: before and after the intervention to evaluate their condition. At both time points, the SLR [25], Modified Bragard's Test [26], Kernig's Test [27], and VAS [28] were administered. Additionally, the VAS was applied during every therapeutic session to monitor manual traction therapy. These assessments took place during the week preceding the commencement of the experimental intervention and the week following its conclusion. They occurred on the same day of the week, ensuring a 48-h rest period before any exercises or sessions the patients may have had. The assessments were conducted in the morning by the same team of evaluators, who were blinded to the experiment. These evaluators were certified physiotherapists with over 10 years of experience in clinical practice and assessments. Furthermore, prior to beginning the study, the team of physiotherapists responsible for assessments conducted a pilot study to train for inter-observer and intra-observer reliability. This involved a sample comprising 10 % of participants who were distinct from those enrolled in the current research. This measure was taken to guarantee data reliability and standardize procedures across the board.

### 2.7 Visual analogue scale (VAS)

The VAS served as a tool for assessing subjective perceptions, particularly pain intensity. Its validity and reliability in assessing pain intensity have been consistently confirmed [28]. We employed a 10-cm horizontal or vertical line, bordered by verbal descriptors at each end representing the extremes of the parameter under evaluation. Participants were instructed to indicate on the line the point that best reflects their perception of the parameter, specifically the intensity of pain. The distance from the end indicating no pain to the participant's mark was then measured and recorded as the score, ranging from 0 to 10. A score of 0 denotes the absence of pain or the parameter being evaluated, while 10 represents the most severe perception imaginable. These anchors offer clear reference points for participants to contextualize their subjective experience, facilitating standardized and quantitative assessments of pain intensity. The VAS was utilized during diagnostic tests as well as during the manual traction sessions.

### 2.8 Straight leg raise test (SLR)

The SLR test was employed to evaluate the integrity of the sciatic nerve and lumbar nerve roots, since it is widely utilized in physiotherapeutic assessments due to its accuracy and reliability [25]. The SLR test was conducted with the patient lying supine on a flat examination table. The hips and knees of the involved leg were positioned neutrally, without abduction or adduction, while the patient's head remained unsupported by a pillow. The physiotherapist securely held the patient's heel in the palm of their hand, while the other hand maintained the knee in extension. With controlled movement, the examiner slowly raised the tested leg up to 90° by flexing the hip, ensuring the knee remained in extension and the limb maintained a neutral position, without external or internal rotation.

The maneuver was considered positive if the patient experienced symptoms reproduced distally to the knee joint, typically between 30° and 70° of hip flexion. To accurately measure the degree of hip flexion, an angular goniometer (Jamar, Hatfield, PA) was utilized at the level of the greater trochanter. The device has been consistently reported as accurate and reliable, as evidenced by previous observations [29]. The VAS score was recorded during the test as the primary outcome of the SLR assessment as well as the angle.

### 2.9 Modified Bragard test

The modified Bragard test [26] was conducted as follows: The patient assumed a supine position on the examination table with both legs fully extended. The test demonstrated sensitivity and discriminative power in assessing patients with nerve root compression, particularly in cases where the straight leg raise test yielded negative results [26]. The examiner initiated the test with the SLR maneuver. In cases where the patient did not manifest any radicular pain or symptoms despite the hip joint being flexed at an angle of 70° (yielding a negative SLR test), the foot was then dorsiflexed forcefully. If pain radiated below the knee upon dorsiflexion, the result of the modified Bragard test was deemed positive. This test operates on the premise that combining hip flexion, knee extension, and dorsiflexion of the ankle enhances the examiner's capacity to elicit signs and symptoms of nerve or ischial root involvement in SLR-negative patients. The VAS score was recorded during the test as the primary outcome of the modified Bragard test assessment.

### 2.10 Kernig's test

The Kernig's Test was employed as a physical examination technique used to assess irritation of the meninges [27]. During the test, the patient lay on their back with their hips and knees flexed. The examiner slowly extended one of the patient's knees. If the maneuver elicited pain or spasms in the hamstring muscles due to stretching of inflamed sciatic nerve roots when the knee was flexed to less than 135°, with the popliteal fossa (the area behind the knee) serving as the inner angle, it indicated a positive Kernig's sign.

The test was based on the hypothesis that the combination of hip flexion and knee extension with dorsal flexion of the ankle would increase the investigator's ability to induce signs and symptoms of nerve/ischial roots in SLR-negative patients [27]. The VAS score was recorded during the test as the primary outcome of the Kernig's Test assessment.

### 2.11 Statistical procedures

No missing cases were reported during the data collection process. The exploratory analysis of the data revealed that the sample did not meet the assumptions of normality (p < 0.05) and homogeneity (p < 0.05) based on the Kolmogorov-Smirnov and Levene tests, respectively. Consequently, non-parametric tests were utilized. Between-group analysis was performed using the Mann-Whitney *U* test, while within-group analysis (post-pre) was conducted using the Wilcoxon signed-ranks test. The effect size was calculated using Cohen's proposed method, utilizing the formula [30]:

(1) $$r=\frac{z}{\sqrt{N}}$$

where *z* represents the value derived from the Mann-Whitney *U* test, and *N* is the number of participants. The interpretation of effect size magnitudes is as follows: 0.0 to 0.1 is considered trivial, 0.1 to 0.3 is small, 0.3 to 0.5 is medium, and anything greater than 0.5 is deemed large [30]. Descriptive statistics were reported as mean and standard deviation. All statistical analyses were carried out using SPSS software (version 29.0, IBM, USA) with a significance level set at p < 0.05.

## 3 Results

Table 1 displays the descriptive statistics for both groups at each assessment time point concerning the main outcomes measured. There were no significant differences between the groups at baseline for any of the outcomes (p > 0.05).

**Table 1:** Descriptive statistics (mean ± standard deviation) of the main outcomes found in both groups at pre and post-intervention moments.

	Experimental Group (n = 30)	Control Group (n = 30)	Difference between groups	Effect size (r)
SLR – Left (°)				
Pre	40.0 ± 10.0	40.5 ± 10.3	U = 429.0; Z = −0.323; p = 0.746	−0.042
Post	46.8 ± 6.9^b^	41.2 ± 9.8	U = 279.0; Z = −2.863; p = 0.004^a^	−0.370
SLR – Right (°)				
Pre	41.0 ± 8.7	40.8 ± 8.9	U = 449.5; Z = −0.008; p = 0.994	−0.001
Post	47.2 ± 6.5^#^	42.0 ± 9.0	U = 283.5; Z = −2.855; p = 0.004^a^	−0.369
Modified Bragard test – Left (°)				
Pre	39.3 ± 9.7	40.0 ± 10.9	U = 419.5; Z = −0.473; p = 0.636	−0.061
Post	45.7 ± 8.1^#^	40.7 ± 10.2	U = 315.0; Z = −2.263; p = 0.024^a^	−0.292
Modified Bragard test – Right (°)				
Pre	40.2 ± 9.4	39.7 ± 9.1	U = 439.5; Z = −0.162; p = 0.871	−0.021
Post	47.2 ± 6.7^#^	41.0 ± 9.4	U = 275.5; Z = −2.997; p = 0.003^a^	−0.387
Kernig's Test – Left (°)				
Pre	40.2 ± 9.1	41.0 ± 9.3	U = 420.5; Z = −0.456; p = 0.648	−0.059
Post	46.8 ± 6.6^#^	42.7 ± 8.3^#^	U = 303.5; Z = −2.483; p = 0.013^a^	−0.321
Kernig's Test – Right (°)				
Pre	41.5 ± 8.2	40.7 ± 8.2	U = 427.0; Z = −0.356; p = 0.722	−0.046
Post	47.0 ± 6.5^b^	43.0 ± 7.6^b^	U = 298.0; Z = −2.574; p = 0.010^a^	−0.332
SLR – Left (VAS, score)				
Pre	1.9 ± 2.8	2.7 ± 2.8	U = 384.0; Z = −1.010; p = 0.313	−0.130
Post	0.4 ± 0.8^b^	1.7 ± 1.9^b^	U = 252.5; Z = −3.305; p < 0.001^a^	−0.427
SLR – Right (VAS, score)				
Pre	2.2 ± 2.4	2.8 ± 2.2	U = 374.0; Z = −1.158; p = 0.247	−0.149
Post	0.3 ± 0.7^b^	1.8 ± 1.6^b^	U = 223.5; Z = −3.838; p < 0.001^a^	−0.495
Modified Bragard test – Left (VAS, score)				
Pre	1.9 ± 1.9	2.6 ± 2.7	U = 391.5; Z = −0.895; p = 0.371	−0.116
Post	0.3 ± 0.6^#^	1.7 ± 1.9^b^	U = 238.5; Z = −3.544; p < 0.001*	−0.458
Modified Bragard test – Right (VAS, score)				
Pre	2.0 ± 2.2	2.6 ± 2.2	U = 381.5; Z = −1.044; p = 0.297	−0.135
Post	0.2 ± 0.6^b^	1.6 ± 1.6^b^	U = 227.5; Z = −3.771; p < 0.001*	−0.487
Kernig's Test – Left (VAS, score)				
Pre	1.9 ± 1.9	2.8 ± 2.7	U = 378.0; Z = −1.101; p = 0.271	−0.142
Post	0.3 ± 0.8^b^	1.9 ± 2.1^#^	U = 246.0; Z = −3.413; p < 0.001*	−0.441
Kernig's Test – Right (VAS, score)				
Pre	2.2 ± 2.4	2.8 ± 2.4	U = 380.0; Z = −1.066; p = 0.287	−0.138
Post	0.3 ± 0.9^b^	1.8 ± 1.8^#^	U = 233.0; Z = −3.680; p < 0.001*	−0.475

VAS: Visual analogue scale; SLR: straight leg raise test.

a significant differences between groups at p < 0.05.

b significant differences within-group (post-pre) at p < 0.05.

Between-group significant differences were found at post-intervention, favoring the experimental group on SLR – Left (°) (p = 0.004; medium effect size), SLR – Right (°) (p = 0.004; medium effect size), Modified Bragard test – Left (°) (p = 0.024; small effect size), Modified Bragard test – Right (°) (p = 0.003; medium effect size), Kernig's Test – Left (°) (p = 0.013; medium effect size) and Kernig's Test – Right (°) (p = 0.010; medium effect size). Additionally, between-group significant differences were found at post-intervention, favoring the experimental group on VAS scores at SLR left (p < 0.001; medium effect size), and right (p < 0.001); medium effect size, Modified Bragard test left (p < 0.001; medium effect size) and right (p < 0.001; medium effect size) and at Kernig's Test left (p < 0.001; medium effect size) and right (p < 0.001; medium effect size).

Within-group analysis (post-pre) on experimental group revealed improved VAS on SLR left (Z = −3.658; p < 0.001; r = −0.668, large effect size) and right sides (Z = −3.650; p < 0.001; r = −0.666, large effect size), Modified Bragard test left (Z = −3.750; p < 0.001; r = −0.685, large effect size) and right sides (Z = −3.643; p < 0.001; r = −0.665, large effect size) and Kernig's Test left (Z = −3.658; p < 0.001; r = −0.668, large effect size) and right sides (Z = −3.644; p < 0.001; r = −0.665, large effect size). Moreover, control group also significantly improved VAS (post-pre) on SLR left (Z = −3.602; p < 0.001; r = −0.614, large effect size) and right sides (Z = −3.624; p < 0.001; r = −0.662, large effect size), Modified Bragard test left (Z = −3.361; p < 0.001; r = −0.614, large effect size) and right sides (Z = −3.685; p < 0.001; r = −0.673, large effect size) and Kernig's Test left (Z = −3.488; p < 0.001; r = −0.637, large effect size) and right sides (Z = −3.511; p < 0.001; r = −0.641, large effect size).

Within-group analysis (post-pre) on experimental group revealed improved angles on SLR left (Z = −3.342; p < 0.001; r = −0.610, large effect size) and right sides (Z = −3.088; p < 0.001; r = −0.564, large effect size), Modified Bragard test left (Z = −3.201; p = 0.001; r = −0.584, large effect size) and right sides (Z = −3.163; p = 0.002; r = −0.577, large effect size) and Kernig's Test left (Z = −3.219; p < 0.001; r = −0.588, large effect size) and right sides (Z = −2.976; p = 0.003; r = −0.543, large effect size). Control group significantly improved angles on Kernig's Test left (Z = −2.428; p = 0.015; r = −0.443, medium effect size) and right sides (Z = −2.565; p = 0.010; r = −0.468, medium effect size), although no significant differences were found at SLR left (Z = −0.966; p = 0.334; r = −0.176, small effect size) and right sides (Z = −1.443; p = 0.149; r = −0.263, small effect size), Modified Bragard test left (Z = −0.966; p = 0.334; r = −0.176, small effect size) and right sides (Z = −1.582; p = 0.114; r = −0.289, small effect size). Thus, clinically, the results indicate that manual traction yields a moderate effect size in ameliorating pain perception and alleviating lumbosacral spine pain attributed to radicular symptoms.

## 4 Discussion

Our research findings indicate that manual traction is significantly effective and superior in alleviating lumbosacral spine pain among patients experiencing radicular symptoms, as opposed to the control group subjected to conventional physiotherapy practices. The incorporation of six manual traction interventions over a two-week period revealed to be sufficient to bring significant improvements in observed angles during the SLR, modified Bragard test, and Kernning's test, along with a reduction in reported pain intensity. Conversely, while the control group experienced a significant reduction in pain intensity, the magnitude of these changes was significantly smaller compared to the experimental group. Moreover, the control group did not demonstrate significant improvements in angles during the modified Bragard test and Kernning's test.

Manual traction significantly benefited the patients' experienced pain intensity. These results corroborate previous studies such as Fritz et al., who compared manual traction alone versus mechanical traction in patients with LBP and nerve root compression. Their findings demonstrated a greater improvement in the patients' condition during the initial two weeks, consistent with the use of manual traction, with no discernible differences between the groups after six weeks of therapy [31]. Additionally, our results align with the study by Beyky et al. [32], which involved a trial with one hundred and twenty-four patients experiencing radicular conflict in the lumbosacral region. This study revealed a reduction in pain, assessed on the VAS, from 6.55 to 2.89 following the application of lumbosacral spine traction [32].

One rationale supporting the potential effectiveness of manual traction lies in its capacity to decompress spinal discs, thereby relieving pressure on the intervertebral foramina, through which spinal nerves exit [33]. This decompression diminishes nerve root compression, thus mitigating radicular pain [21]. It is postulated that the mechanoreceptors present in the muscular and ligamentous structures of the disc are activated by stretching, potentially inhibiting pain impulses [34]. Manual traction can elongate taut muscles and fascia, enhancing flexibility and reducing muscle spasms, thereby contributing to pain alleviation [33]. Furthermore, traction stimulates the release of endorphins, further augmenting relief [35].

Our study demonstrated significant improvement in the angles measured by the goniometer during the tests following manual traction. Conversely, within the control group, the observed differences were mostly nonsignificant and consistently significantly inferior to those observed with manual traction in all cases. Our results are consistent with previous findings, such as those of Khan et al. [36], which showed that two weeks of manual traction for cervical radiculopathy significantly improved range of motion and reduced pain. However, our results contradict the study conducted by Young et al., which did not observe significant advantages of manual traction compared to regular active physiotherapy modalities regarding function [37].

The efficacy of manual traction over the control group (only exposed to diadynamic currents, light therapy with a Sollux lamp, laser therapy, and magnetotherapy) in improving angles on SLR test, modified Bragard test, and Kerning's test could be attributed to several theoretical reasons. Manual traction involves a hands-on approach where a practitioner applies controlled force to manipulate joints and soft tissues, potentially facilitating the realignment of anatomical structures and relieving nerve compression [38]. This may directly target the underlying mechanical factors contributing to limited range of motion in these tests [39]. Additionally, manual traction can stimulate proprioceptors and mechanoreceptors in muscles and joints, eliciting neuromuscular responses that promote relaxation [33], increased flexibility, and improved muscle function. In contrast, while modalities like diadynamic currents, light therapy, laser therapy, and magnetotherapy may offer benefits such as pain relief and tissue healing through various mechanisms like modulation of inflammation and promotion of cellular repair, their effects on joint mobility and neural tension may not be as direct or pronounced as manual traction. Therefore, the observed superiority of manual traction in this context could be explained by its ability to address both mechanical and neurological aspects of musculoskeletal dysfunction, leading to more significant improvements in test angles.

While our study was randomized and controlled, it is not lacking limitations. For instance, we were unable to conduct a follow-up to determine the duration of effects after therapy cessation. Additionally, we did not compare manual therapy with mechanical therapy. Moreover, blinding during the allocation of patients to study groups was not implemented. Future research should aim to compare both modalities and assess their effects relative to other forms of active exercise. Furthermore, ensuring blinding is imperative to mitigate bias in future studies. From a methodological perspective, manual traction may introduce variability in the applied forces, influenced by individual participant characteristics. Therefore, prioritizing future standardization of techniques is crucial for facilitating a more personalized analysis. Despite these limitations, our study revealed the efficacy of manual traction in significantly reducing both pain intensity and functional impairment among patients with radicular symptoms. It shows to be significantly superior to the conventional therapy utilized by the control group. Thus, manual traction can be considered a safe and effective option for this patient population. Furthermore, given that participants did not report any adverse effects during the experimental procedure, it is feasible to assert the safety of the approach for the participants.

## 5 Conclusions

In conclusion, our study shows that manual traction interventions administered over a two-week period are significantly more effective than conventional physiotherapy practices in alleviating lumbosacral spine pain among patients with radicular symptoms. The incorporation of six manual traction sessions led to notable improvements in observed angles during the SLR, modified Bragard test, and Kernning's test, accompanied by a significant reduction in reported pain intensity. Conversely, the control group, while experiencing some reduction in pain intensity, did not exhibit significant improvements in test angles, highlighting the superiority of manual traction in addressing radicular symptoms and enhancing functional outcomes.

## Consent for publication

Not applicable.

## Ethical aspects

The study design received approval from the Ethics Committee of Wrocław University of Health and Sport Sciences (NKBBN1/2023).

## Informed consent

written inform consent was provided.

## Funding

Nothing to declare.

## Data availability

The data is available upon reasonable request to the corresponding author.

## CRediT authorship contribution statement

**Rafał Studnicki:** Writing – review & editing, Writing – original draft, Methodology, Formal analysis, Data curation, Conceptualization. **Piotr Szymczyk:** Writing – review & editing, Writing – original draft, Investigation, Data curation. **Tomasz Adamczewski:** Writing – review & editing, Writing – original draft, Methodology, Conceptualization. **Karolina Studzińska:** Writing – review & editing, Writing – original draft, Conceptualization. **Rita Hansdorfer-Korzon:** Writing – review & editing, Writing – original draft, Resources, Investigation. **Ana Filipa Silva:** Writing – review & editing, Writing – original draft. **Adam Kawczyński:** Writing – review & editing, Writing – original draft, Supervision.

## Declaration of competing interest

The authors declare that they have no known competing financial interests or personal relationships that could have appeared to influence the work reported in this paper.
